# Good Character at College: The Combined Role of Second-Order Character Strength Factors and *Phronesis* Motivation in Undergraduate Academic Outcomes

**DOI:** 10.3390/ijerph18168263

**Published:** 2021-08-04

**Authors:** Jorge L. Villacís, Jesús de la Fuente, Concepción Naval

**Affiliations:** 1 Department of Research Theory and Methods in Education and Psychology, School of Education and Psychology, University of Navarra, 31008 Pamplona, Spain; jdlfuente@unav.es (J.d.l.F.); cnaval@unav.es (C.N.); 2 Department of Psychology, School of Psychology, University of Almería, 04120 La Cañada de San Urbano, Spain

**Keywords:** positive psychology, moral development, practical wisdom, school-work transition

## Abstract

A renewed interest in the study of character and virtue has recently emerged in the fields of Education and Psychology. The latest research has confirmed the association between virtuous consistent behaviours and academic positive outcomes. However, the motivational dimension of character (the intentions underlying the patterns of observed behaviours) has received little attention. This research aims to extend the knowledge on this topic by examining the predictive relationships between the behavioural and motivational dimensions of character, with reference to academic engagement, career self-doubt and performance of Spanish university students. A total of 183 undergraduates aged 18–30 (142 of whom were women) from the north of Spain completed specific parts of self-report questionnaires, including the Values in Action VIA-72, a Spanish translated and validated version of the Moral Self-Relevance Measure MSR, and the Utrecht Work Engagement Student Scale UWES-S9. The collected data were analysed using Structural Equation Modelling. The behavioural dimension of character (character strength factors of caring, self-control and inquisitiveness) showed positive associations with academic engagement and performance. The motivational dimension of character (*phronesis* motivation), was negatively related to career self-doubt. For the first time, the present study has provided support for the contribution of both dimensions of character to undergraduate academic outcomes.

## 1. Introduction

In the past few decades, social scientists and educators have demonstrated a renewed interest in the study of character and virtues [1,2,3,4]. Character is considered a key element for thriving at times of adversity [5], and is a constitutive part of human flourishing and work-related well-being [6]. A wide variety of public and private initiatives have crystallized these ideas by including moral and character education in the school curriculum [3].

Sometimes that inclusion has been explicit by adding new subjects to the compulsory curriculum, whilst at other times it has been conducted through offering optional activities to school and high school students [1] (p. 8). These initiatives also differ in their theoretical grounding, going from explicitly moral and virtue-focused (e.g., Neo-Aristotelian Character Education [1]), to more eclectic approaches aimed at promoting life skills and socio-emotional competencies (e.g., Positive Youth Development and Socio-Emotional Learning [3]). In the recent time, character-focused interventions have also been developed for young people in university settings [7,8]. Among the factors that help to explain the new interest in character and its education, some authors have suggested the necessity to prevent harmful conduct (i.e., addiction, bullying, depression) and promote well-being in educational contexts [3], as well as address complex societal issues such as the value-gap opened by secularisation and multiculturalism [1] (p. 4).

Although a resurgence of this study of character in education and psychology is evidenced, some questions remain unresolved. First, most research on character education has been focused on primary and secondary school settings [9]. Little is known about how character development occurs during emerging adulthood and what the best strategies to foster its development are. Second, there are complex conceptual issues regarding how virtues and character are measured [4,10]. For some authors, the consistency of virtue-related behaviours is sufficient to indicate the presence of character [11,12]. However, other researchers have suggested that moral motivation (the intentions or reasons underlying the observed behaviours) is also necessary for possessing virtue [13,14]. By shedding light on these questions, it is believed that more reliable educational and psychological interventions can be encouraged that can promote well-being through character development. The current study aims to provide evidence about the contribution of both aspects of character (behavioural and motivational) for positive academic outcomes of young university students.

### 1.1. Character Strengths as Predictive Factors of Academic Engagement, Career Self-Doubt and Academic Performance

Within psychology, one of the most extended approaches to the study of virtues and character is the one proposed by positive psychology [15]. This approach states that virtues are “the core characteristics valued by moral philosophers and religious thinkers”, and character strengths are “the psychological ingredients—processes or mechanisms—that define the virtues” [12] (p. 13). Character strengths are considered morally valued personality traits [12] (p. 19). Described as “the social science equivalent of virtue ethics” [12] (p. 89), this model of character is composed of 24 character strengths grouped into several virtues. Peterson and Seligman [12], the original developers of the positive psychology character strength model, proposed a list of six overarching virtues grouping the 24 subordinated character strengths: (1) wisdom—with the strengths of creativity, curiosity, judgement, love of learning and perspective; (2) courage—including the strengths of bravery, perseverance, honesty and zest; (3) humanity—grouping love, kindness and social intelligence; (4) justice—including teamwork, fairness and leadership; (5) temperance—with the strengths of forgiveness, humility, prudence and self-regulation; and (6) transcendence—with appreciation of beauty, gratitude, hope, humour and spirituality.

Refinements in the classification of character strengths around virtues were anticipated by Peterson and Seligman [12] (p. 31). Subsequent empirical research has suggested that a three-virtue model can consistently group the 24 character strengths [16,17,18]. The three-virtue model has been argued as a reliable latent structure for the character strengths across studies [16,17,18]. Furthermore, this model has been recommended as a psychologically and culturally meaningful model to encourage research on virtue [17]. The three virtues, which can be described as second-order factors of the 24 character strengths, have been named caring, self-control and inquisitiveness. Caring is a virtue that includes the strengths involved in interpersonal relationships (e.g., gratitude, kindness and love) [17]. Self-control comprises the strengths that allow effective functioning in the world (e.g., perseverance, prudence and self-regulation) [17]. Inquisitiveness is formed by the strengths that reflect intellectual endeavours (e.g., creativity, love of learning and perspective) [17]. These three virtues are claimed to capture the three targets of virtuous actions: others, the self and the physical world [17,19].

The possession and use of virtues and character strengths have been considered pathways to achieve positive outcomes across different life domains including the educational one [20]. Building and strengthening good character has been considered an essential goal for education of children and young people [21]. In a recent literature review, character strengths were compared to the 21st century competencies for thriving in life, work and education proposed by the American National Research Council [2]. Empirical research has also explored which character strengths are the most relevant for the educational domain. According to the layperson’s perspective, the strengths of curiosity, judgement, love of learning, perseverance and self-regulation were highly relevant to flourishing in education [22]. In the following paragraphs, a summary of previous research on the relationships between character strengths and the academic outcomes of engagement, career self-doubts and performance will be presented. These outcomes are thought to represent indicators of adaptation and well-being in the educational domain.

Academic engagement can be defined as “a positive, fulfilling, work-related state of mind characterized by vigour, dedication and absorption ... applied to the activities students perform” [23]. It expresses the positive affectivity that may accompany academic task fulfilment (e.g., attending lectures, doing homework or studying for a test). Past studies have found associations between some of the strengths that comprise the caring factor and academic engagement. Gratitude, love, leadership and kindness strengths showed positive correlations with academic engagement in a sample of medicine undergraduates [24]. In a study with adolescents, the fairness, teamwork, leadership, forgiveness and kindness strengths showed positive associations with academic engagement [25]. In addition, kindness perception in high school settings has been related to academic engagement [26]. In the case of self-control, several strengths that group together in this factor have been related to academic engagement. Perseverance and self-regulation have shown positive relationships to academic engagement in university students [24]. Furthermore, a study that grouped the self-control strengths into a single factor showed a positive association between this factor and the academic engagement of undergraduates [27]. With regard to inquisitiveness, certain strengths that belong to this factor have been associated with academic engagement. Curiosity, creativity and love-of-learning strengths have shown positive relationships to academic engagement in medicine undergraduates [24]. In a study that grouped the inquisitiveness strengths into a single factor, there was a positive association between this factor and academic engagement [27].

Career self-doubt, in contrast to academic engagement, can be considered an indicator of negative emotionality in the educational domain. Defined as the extent to which a student is uncertain about their career choice [28], the career self-doubt construct has been considered unfavourable for adolescents’ and young adults’ well-being [28,29]. Higher levels of career self-doubt have been associated with low levels of purpose in life and eudaimonic well-being, as well as with higher anxiety and depression [29]. Past theoretical studies have emphasized the positive role of character strengths in the vocational development of adolescents and young adults [30,31]. The possession and use of character strengths are believed to be positive resources that help young people explore and commit to meaningful career alternatives and avoid further indecision [31,32,33]. Regarding caring, some strengths that pertain to this factor have been associated with career self-doubt. The strengths of love, measured with attachment scales [34,35], and social intelligence, assessed with emotional intelligence scales [36,37,38], have shown negative associations with career self-doubt in university students. With regard to self-control, various strengths that belong to this factor have been related to career self-doubt. Prudence, measured with conscientiousness subscales [37,38,39,40,41], and self-regulation strengths have shown negative associations with career self-doubt in undergraduates [42]. Concerning inquisitiveness, several strengths that form this factor have been associated with career self-doubt. Curiosity, measured with openness subscales [43,44,45], and love of learning have shown negative associations with career self-doubt in young adult students [46].

Academic performance, also referred to as academic achievement, expresses any identifiable success in the areas of scholarship or disciplined study [47]. Previous research has examined the association between caring strengths and academic performance. Kindness, forgiveness and fairness have been significantly associated with the academic scores of undergraduates [48]. Moreover, fairness has shown a positive association with self-reported GPA [49]. Among high school students, the caring factor has been positively associated with final exam grades [50]. Regarding self-control, this factor and some of its strengths have been related to academic performance. Studies using self-control scales have demonstrated a positive association between this construct and academic achievement [51,52,53]. In addition, the self-control factor has also shown a positive association with final exam grades in high school students [50]. Among university students, self-regulation, perseverance and prudence [49], as well as honesty and humility, [48] have demonstrated positive associations with academic scores. Concerning inquisitiveness, several strengths that belong to this factor have presented associations with academic performance. The strengths of curiosity and perspective have shown positive associations with self-reported GPA in university students [49]. In addition, love of learning has been positively correlated to academic performance in undergraduates [48,49] and adolescents [54,55]. Finally, the inquisitiveness factor has shown a positive association with final exam grades in high school students [50].

### 1.2. A Gap in the Conceptualization of Character Strengths and the Phronesis Construct

Although character strengths are considered to be trait-like measures of the moral virtues [56], several authors have raised concerns about this concept. A key aspect of the classic notion of virtue, i.e., its moral intentionality or motivation, has been argued to be absent from the character strength model [13,14,57]. As with commonly used personality measures (e.g., Big Five self-report questionnaires), a high score in a specific trait, e.g., self-regulation, can be demonstrated by both the self-disciplined criminal and the conscientious Mother Teresa [14] (pp. 79–83). It is not possible to infer the reasons or motives behind the behavioural traits measured [13]. The relevance of considering the internal aspects of morality in character strength research has been also acknowledged by the latest empirical research. Recent studies have examined how moral values can be expressed through specific character strengths (e.g., gratitude [58]), and how character strengths differ in the degree to which laypersons consider them to be morally valued [59]. Based on this research, it is argued that the combination of the character strengths model with a construct describing moral motivation would permit a more holistic understanding of the classical notions of virtue and character.

There are several constructs in moral psychology that can be used to account for moral motivation [60]. Among such constructs, the meta-virtue of *phronesis*, or practical wisdom, has recently attracted the attention of character researchers [56,60,61,62]. Rooted in the virtue ethics tradition, *phronesis* is defined as an “intellectual meta-virtue of holistic, integrative, contextual, practical reflection and adjudication about moral issues, motivating moral action” [63] (p. 8). This meta-virtue is thought to promote the effective application of the virtues according to the specificity of a given situation [56]. In addition, *phronesis* is said to provide moral motivational force to the agent, aligning their emotional response with their perception of the required good [60].

In recent works, the *Jubilee Centre for Character and Virtues* has offered empirical evidence for an Aristotelian *Phronesis* Model [60,63,64]. According to this model, the meta-virtue of *phronesis* comprises four psychological components. These components are: (1) the constitutive function, or the cognitive ability to perceive the ethically relevant features of a situation; (2) the integrative function, which balances the different components of a good life in dilemmatic situations; (3) the blueprint component, or an overall sense of the kind of things that matter for a flourishing life; and (4) the emotional regulation component, which brings emotional responses into line with an ethical understanding of the situation [63]. A *Phronesis* Inventory is currently at its design phase by the researchers of the Jubilee Centre [63]. However, some measures derived from moral psychology have been proposed as a starting point to operationalize each component of the *phronesis* meta-virtue [60,64]. For instance, moral reasoning measures (e.g., Defining Issues Test [65]) seem to be close to the integrative function of *phronesis*; moral identity measures (e.g., Moral Self-Relevance Measure [66]) can be used as indicators of the blueprint component; and empathy measures (e.g., Interpersonal Reactivity Index [67]) are thought to be partly expressing the emotion-regulatory component. Following suggestions of current empirical virtue research [4], we consider that the inclusion of the *phronesis* construct within the character strengths model is in line with current efforts aiming to translate the classic notion of virtue into a scientific model of character.

### 1.3. The Present Study. Aims and Hypotheses

Acknowledging the state of current research in virtues and character strengths, and the possibilities offered by the emerging *phronesis* construct, the present study proposes a first examination of how these two constructs can contribute to positive academic outcomes of young university students. On the one hand, current research has shown how different character strengths are related to academic engagement, career self-doubt and performance. However, not in all the cases were the same strengths related to the academic outcomes reviewed in the literature. More empirical research appears to be needed to help establish the set of character strengths that can best contribute to different positive academic outcomes. On the other hand, no research—to our knowledge—has included the moral motivational aspects of character (for instance, using the *phronesis* motivation construct) as an additional predictor of academic outcomes. A study intending to explore this facet will be of great relevance, especially for the young adult population. This developmental stage has been suggested as adequate for the acquisition of the *phronesis* meta-virtue according to theoretical analyses [68] (p. 9).

To fill these gaps, the current study aims to examine the predictive relationships between character strength factors and *phronesis* motivation, regarding academic engagement, career self-doubt, and performance in a sample of Spanish undergraduates. By doing this, knowledge about the specific contribution of the behavioural (character strength factors) and motivational (*phronesis* motivation) dimensions of character will be expanded. Since this study includes both dimensions of character (behavioural and motivational) for the first time, we chose to use the three virtues model [16] to simplify the analysis. This model has been validated and found to be consistent in representing the global aspects of the behavioural dimension of character [16,17,69]. In addition, the three virtues of this model are claimed to capture the three targets of virtuous actions: others, the self, and the physical world [17,19]. Lastly, by studying this topic in undergraduate students, it is possible to begin to address the breach in the literature on character development in young adults. To achieve the principal goal of this study, we established the following specific objectives and hypotheses:

(1) Summarize the characteristics of the participants regarding character strengths, *phronesis* motivation, academic engagement, career self-doubt and performance.

**Hypothesis** **1** **(H1).** *Participants will exhibit similar scalar scores levels at the measurement instruments when compared to past studies.*

(2) Establish the predictive relationships of character strength factors as regards academic outcomes.

**Hypothesis** **2** **(H2).** *Character strength factors will show statistically significant predictive relationships with regard to academic engagement, career self-doubt and performance.*

(3) Describe the differences in the pattern of predictive relationships between character strength factors and academic outcomes when *phronesis* motivation is included as an additional predictive factor.

**Hypothesis** **3** **(H3).** *Phronesis motivation will show statistically significant predictive relationships with regard to academic engagement, career self-doubt and performance, after controlling for character strength factors.*

**Hypothesis** **4** **(H4).** *The inclusion of phronesis motivation will increase the explained variance of the academic outcomes measured in this study when compared to a model with character strength factors as the only predictors.*

## 2. Materials and Methods

### 2.1. Participants

The sample consisted of 183 undergraduate students (142 women) from a university located in the north of Spain. Their mean age was 20.1 years (SD = 1.92, range 18–30 years). Half of the sample was composed of students of Psychology (49.7%), while the remainder were studying Communications (34.4%), Bio-Chemistry (10%), and other degrees (5.9%). Almost half of the participants were in their first year (47.5%), followed by a large group in their fourth and final year (33.9%). The sampling was incidental and non-probabilistic. The high proportion of women in the sample can be explained partly due to the high presence of females studying for humanistic degrees, as for instance Psychology.

### 2.2. Instruments

#### 2.2.1. Character Strength Factors

The *Values in Action Inventory of Strengths short form VIA-72* [12] is a 72-item self-report questionnaire assessing the 24 character strengths proposed by Peterson and Seligman [12]. Each character strength is measured with three items that follow a 5-point Likert-style response system (from 1 = “Very Much Unlike Me” to 5 = “Very Much Like Me”). Examples of items are: “I really enjoy doing small favours for friends” (kindness) and “I am a highly disciplined person” (self-regulation). In the present study, we utilized only nine of the character strength scales to form the 3-virtues compound scales proposed by Berger and McGrath [70]: caring (formed by the character strengths of gratitude, love and kindness), self-control (composed of perseverance, prudence and self-regulation strengths), and inquisitiveness (formed by perspective, creativity and love-of-learning strengths). The second-order confirmatory model (using the nine character strengths as indicators) showed a good fit with the data (*X*^2^ = 35.0, *p =* 0.07, *df* = 24, *X*^2^*/d f* = 1.46, CFI = 0.96, TLI = 0.94, RMSEA = 0.05, and SRMR = 0.06). Compound reliability of the 3-virtues scales showed optimal values (caring, ω = 0.88; self-control, ω = 0.82; inquisitiveness, ω = 0.85).

#### 2.2.2. Phronesis Motivation

For measuring *phronesis*, we used the Darnell et al. [60] Aristotelian *Phronesis* Model. Although four components of *phronesis* were proposed, we focused on two of them in this research: emotional regulation and blueprint. The following measurement instruments were employed:

(a) The *Interpersonal Reactivity Index IRI* [67] is a self-report questionnaire designed to assess the moral emotion of empathy. The IRI is composed of four sub-scales: perspective-taking, empathic concern, fantasy and personal distress. In the present study, we utilized only the emphatic concern 7-item scale. An item example of this scale is “I often have tender, concerned feelings for people less fortunate than me”. IRI items follow a 5-point Likert-type response system (from 1 = “Does not describe me well” to 5 = “Describes me very well”). A Confirmatory Factor Analysis (CFA) with two separate factors for positively and negatively keyed items showed adequate fit to the data (*X*^2^ = 14.18, *p* = 0.36, *df* = 13, *X*^2^*/d f*= 1.09, CFI = 0.99, TLI = 0.99, RMSEA = 0.02, and SRMR = 0.03). Internal consistency of the empathic concern scale was also adequate with the current sample (α = 0.74).
(b) The *Moral Self-Relevance Measure MSR* [66] is a self-report questionnaire intended to evaluate moral identity. The questionnaire is divided into two parts. The first part is composed of 16 items that follow a 5-point Likert-type response system (from 0 = “Not important to me” to 4 = “Extremely important to me”). In this part, respondents are asked to rate the importance of moral (e.g., kindness) and non-moral qualities (e.g., creativity) for their sense of self. In the second part, respondents have to choose 8 out of 32 positive qualities (including both moral and non-moral traits). The questionnaire was scored following the procedure established by its authors, see [66], counting only those responses in which participants included moral qualities. The total score ranged from 0 to 32. Since this is the first adaptation of the MSR to Spanish, the guidelines of the International Test Commission [71] and the recommendations of Muñiz, Elosua, and Hambleton [72] were followed. Forward translation design was applied [73]. A panel formed by three Spanish native speakers with expertise in moral education translated the MSR from English to Spanish. Afterwards, a professional translator, with fluency in both English and Spanish, revised the previously translated version and suggested some minor modifications. A pilot test (n = 21) was conducted with a separate sample of undergraduate students to ensure the translated questionnaire was correctly understood. A Confirmatory Factor Analysis with two first-order factors (items referred to honesty or kindness traits), and a second-order factor showed adequate fit to the data (*X*^2^ = 27.74, *p* = 0.27, *df* = 24, *X*^2^*/d f*= 1.16, CFI = 0.98, TLI = 0.97, RMSEA = 0.03, and SRMR = 0.04). Internal consistency and compound reliability were adequate in the current sample (α = 0.70, ω = 0.79).
(c) The *Contingencies of Self-Worth Scale CSW* [74] is a self-report measure assessing seven sources of self-esteem. Participants in this study completed only the 5-item Virtue subscale. An example item is “My self-esteem depends on whether I follow my moral/ethical principles or not”. The items follow a 7-point Likert-type response system (from 1 = “strongly disagree” to 7 = “strongly agree”). A CFA of the Virtue subscale, which included two estimated correlations between error terms due to methodological reasons, showed adequate fit to the data (*X*^2^ = 14.18, *p* = 0.36, *df* = 13, *X*^2^*/d f*= 1.09, CFI = 0.99, TLI = 0.99, RMSEA = 0.02, and SRMR = 0.03). The Virtue subscale showed satisfactory internal consistency with the current sample (α = 0.74).

A Confirmatory Factor Analysis, which included the three instruments used for measuring *phronesis*, showed adequate fit to the data (*X*^2^ = 214.23, *p <* 0.05, *df* = 180, *X*^2^*/df* = 1.19, CFI = 0.95, TLI = 0.94, RMSEA = 0.03, SRMR = 0.06). In such analysis, items were used as indicators, and they were grouped into four first-order factors (items from the Interpersonal Reactivity Index were separated into two factors depending on whether they were positively or negatively keyed), and one *phronesis* second-order factor.

#### 2.2.3. Academic Engagement

The *Utrecht Work Engagement Student Scale UWES-S9* [75] is a 9-item scale designed to measure engagement and positive affectivity in academic settings. The items can be grouped into three subscales: vigour, dedication and absorption. In the current study, we employed the vigour and dedication subscales only. Examples of items are: “My work as a student makes me feel full of energy” (vigour) and “I am enthusiastic about my career” (dedication). Items follow a 7-point Likert-type response system (from 0 = “Never” to 6 = “Always/Every day”). A CFA using the 9 items as indicators and three first-order factors showed adequate fit to the data in the current sample (*X*^2^ = 38.08, *p <* 0.05, *df* = 24, *X*^2^/*df* = 1.59, CFI = 0.98, TLI = 0.96, RMSEA = 0.06, and SRMR = 0.04). In the present study, Cronbach’s Alpha was 0.79 for the vigour and 0.84 for the dedication subscale.

#### 2.2.4. Career Self-Doubt

The *Vocational Identity Status Assessment VISA* [28] is a 30-item self-report measure of the six vocational processes that constitute vocational identity. Each process is measured with a 5-item subscale. In the present study, we employed the Career Self-Doubt subscale. This subscale assesses the degree of uncertainty about the chosen career and joining the labour market. It has been associated with negative psychosocial functioning and negative affectivity, according to previous research [29]. An item example of this scale is “I doubt I will find a career that suits me”. The items follow a 5-point Likert-type response system (from 1 = “strongly disagree” to 5 = “strongly agree”). In this research, the Career Self-Doubt subscale from the VISA instrument was translated into Spanish following the same procedure described for the Moral Self-Relevance Measure in this study. First-order confirmatory factor analyses of the Career Self-Doubt subscale showed adequate fit to the data in this study (*X*^2^ = 6.55, *p* = 0.26, *df* = 5, *X*^2^*/df* = 1.31, CFI = 0.99, TLI = 0.97, RMSEA = 0.04, and SRMR = 0.03). Internal consistency of the Career Self-Doubt subscale was also adequate with the current sample (α = 0.72).

#### 2.2.5. Academic Performance

A single item adapted from the *International Self-Report Delinquency Questionnaire 3 ISRD-3* [76] was employed to assess self-reported academic performance. To the question “How well are you doing in class?”, participants responded using a seven-point Likert Scale answer system (from 1 = “Poorly, I’m probably one of the worst in my class” to 7 = “Excellently, I’m probably one of the best in my class”).

Participants also completed a number of other measures, including the remaining scales from some of the reported instruments. Those measures were not of central interest to the objectives of the present study and formed part of a research project about the moral and vocational development of young adults still in process.

### 2.3. Procedure

Students were invited to collaborate in this research after class periods. Participation was voluntary. Students who decided to participate signed a digital consent form and completed an anonymous online survey. The survey included the measurement instruments, some additional scales and socio-demographic questions. Participants were free to complete the survey in a university classroom (using their own electronic devices) or at home. More than 80% of the participants chose to complete the survey in the classroom. Three gift cards with EUR 50 credit for the campus stationery shop were raffled among all the participants as a form of compensation. The research protocols were approved by the Committee on Ethics in Research (University of Navarra, ref. 2019.165), and met all the requirements of the Code of Ethics in Psychology and the Spanish Data Protection Act.

### 2.4. Data Analysis

An ex post facto and transversal design were used. For *specific objective 1*, descriptive statistics and bivariate Pearson correlations were computed using SPSS version 23 (IBM, Armonk, N.Y., USA). To assess multivariate normality, we calculated Mardia’s multivariate normality test using the MVN package [77] in R version 4.0.2. According to this test, multivariate normality is probed when both p-values of skewness and kurtosis coefficients are greater than 0.05. For the *specific objectives 2 and 3*, the relationships between the study variables were inspected using a Structural Equation Modelling (SEM) framework. For the SEM analyses, we utilized the lavaan package [78] in R version 4. 0. 2 (R Foundation for Statistical Computing, Vienna, Austria). We used robust Maximum Likelihood (MLR) as the estimation method. This method has been suggested when the variables are not normally distributed and the sample size is small [79]. Missing values were treated using the full information maximum likelihood (case-wise) approach. Model fit was evaluated taking into account traditional criteria [80,81]: Comparative Fit Index (CFI), and the Tucker–Lewis index (TLI) with values of over 0.90 as indicative of adequate fit; the Root Mean Square Error of approximation (RMSEA) with a cut-off value close to 0.06, and the Standardized Root Mean Square Residual (SRMR) with a value less than 0.08 suggesting a satisfactory solution.

## 3. Results

### 3.1. Descriptive Analyses

Descriptive statistics and bivariate Pearson correlations among all the study variables are shown in Table 1. With reference to mean statistics, character strengths expressing caring behaviours (e.g., gratitude, love, kindness) showed higher scores in comparison with the rest of the strengths. The love-of-learning strength presented the lowest score. Between the two academic engagement subscales, dedication exhibited the highest mean score. In the case of both career self-doubt and self-reported academic performance, the mean score reflected the tendency of participants to locate themselves at the low-intermediate part of these scales. When the variable distribution shapes were examined, we observed that except for love of learning, career self-doubt and academic performance, all the remainder presented negative skewness. This implies that the observed values in most of the variables tended to be concentrated in the upper/higher segment of their corresponding scales. Regarding kurtosis, six of the sixteen variables showed values higher than 0.30, and five presented values lower than −0.30. For some of these variables, the majority of the observed values tended to be concentrated in a specific section of the measurement scale (positive kurtosis), whilst for others, responses were more or less homogeneously distributed along the measurement scale (negative kurtosis).

Regarding bivariate correlations, there were significant associations between most of the character strengths or *phronesis* scales and the academic variables. Eight of the nine character strengths were significantly related to the vigour dimension of engagement, and six were associated with the dedication dimension. Three character strengths were associated with career self-doubt. Lastly, only two strengths (love of learning and perseverance) were correlated to academic performance. Regarding *phronesis* motivation, all of these scales showed significant associations with both engagement and self-doubt. However, none of the *phronesis* motivation scales were related to academic performance. The size of the correlations varied from 0.15 to 0.32.

### 3.2. Predictive Relationships of Character Strength Factors with Respect to Academic Engagement, Career Self-Doubt, and Performance

Some preliminary analyses were conducted to test the suitability of the data for SEM. The Kaiser–Meyer–Olkin measure of Sampling Adequacy (KMO) and Bartlett’s sphericity test were computed for the variables in this study (9 character-strength scale scores, 3 *phronesis* scale scores, 6 academic engagement items, 5 career self-doubt items and 1 academic performance item). Both tests indicated that the included variables fulfilled the factorization prerequisites: KMO = 0.80; Bartlett’s sphericity test: *X*^2^ = 1387.54, df = 276, *p <* 0.000. According to these tests, 80% of the variance of the included variables can be explained by underlying factors. In addition, the matrix of correlations between the included variables showed them to be suitable for structure detection. Regarding Mardia’s multivariate normality tests, both skewness (*X*^2^ = 3473.93, *p <* 0.000) and kurtosis (Z = 7.36, *p <* 0.000) coefficients were significant. This indicated that the data were not multivariate normally distributed. For this reason, the robust Maximum Likelihood (MLR) estimator was employed in all the following SEM analyses.

The first model we adjusted (M1) included the three character-strength factors of caring, inquisitiveness and self-control, predicting the two dimensions of academic engagement (vigour and dedication), career self-doubt and self-reported academic performance. We allowed each character-strength factor to predict all the academic variables. In total, 12 regression path coefficients were estimated for this model. As it is presented in Table 2, the model exhibited an adequate fit to the data. After inspecting the regression paths of the M1 model, we found only three significant regression coefficients (*p <* 0.05): The caring factor showed to be a significant predictor of vigour (standardized coefficient = 0.27; *p <* 0.05) and career self-doubt (standardized coefficient = –0.25; *p <* 0.05). In addition, the inquisitiveness factor significantly predicted academic performance (standardized coefficient = 0.30; *p <* 0.05). Regarding the remaining regression paths, we observed seven coefficients with p-values over 0.20 and two with under 0.20.

Maintaining the previous structure, we adjusted a second model in which some of the non-significant regression paths were omitted. In this new model (M2), we constrained at 0 the seven regression paths that showed p-values greater than 0.20 in the previous model. Fit statistics of the M2 model are shown in Table 2. Of the five estimated regression paths of this second model, four were significant (p < 0.05) and one was marginally significant. The caring factor continued to be a significant predictor of vigour (standardized coefficient = 0.40; p < 0.001) and career self-doubt (standardized coefficient = −0.26; *p <* 0.05), and also predicted dedication (standardized coefficient = 0.29; p < 0.05). The inquisitiveness factor was a significant predictor of academic performance (standardized estimate = 0.26; *p < 0*.01). The only marginally significant predictive factor was self-control with regard to dedication (standardized coefficient = 0.23; p = 0.10).

### 3.3. Predictive Relationship between Character Strength Factors and Phronesis Motivation Regarding Academic Engagement, Career Self-Doubt and Performance

In the following analyses, we included *phronesis* as an additional predictor of academic outcomes. Models were formed by the three character strength factors (caring, inquisitiveness and self-control) and the *phronesis* motivation factor as predictors of academic engagement (including vigour and dedication subdimensions), career self-doubt and academic performance. In the M3 model, each of the character strength factors and the *phronesis* construct were predictors of all four academic outcomes’ factors. In total, 16 regression paths were estimated for this model. Fit statistics are presented in Table 2. The M3 model showed an adequate fit to the data. Of the 16 regression paths, three coefficients were found statistically significant (p < 0.05). These coefficients were *phronesis* predicting dedication (standardized coefficient = 0.59; p < 0.01) and career self-doubt (standardized coefficient = −0.71; *p <* 0.01), and inquisitiveness predicting academic performance (standardized coefficient = 0.31; p < 0.05). Regarding the other 13 regression paths, there were eight coefficients with p-values greater than 0.20, and two with p-values less than 0.20.

**Figure 1:**
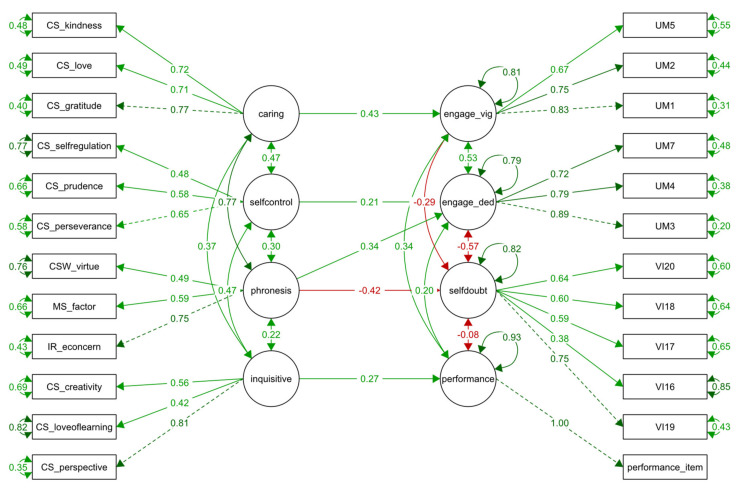
Model M4. Character Strength Factors and *Phronesis* Motivation as predictors of academic engagement (engage_vig = vigour dimension; engage_ded = dedication dimension), career self-doubt (self-doubt) and academic performance (performance), SEM diagram of standardized estimates. Coefficients in green represent positive values (>0), and coefficients in red are negative values (<0).

We adjusted a final model (M4), which included only significant regression paths (*p <* 0.05). To select the paths, we included those that were significant and marginally significant in the previous M3 model. In total, five regression paths were estimated in this model. The M4 model exhibited adequate fit to the data as shown in Table 2. The five regression paths of this model were shown to be significant: caring predicting vigour (standardized coefficient = 0.43; p < 0.001), *phronesis* predicting both dedication (standardized coefficient = 0.35; *p < 0*.01) and career self-doubt (standardized coefficient = −0.42; *p <* 0.001), self-control predicting dedication (standardized coefficient = 0.21; *p <* 0.05) and inquisitiveness predicting academic performance (standardized coefficient = 0.27; *p <* 0.01). A diagram of this model is presented in Figure 1.

Lastly, we compared the amount of predicted variance in the latent factors of the models M2 and M4. As presented in Table 3, there was an increment in the R-Square coefficient for three of the outcomes in model M4. In this model, more variance of the dedication, vigour and career self-doubt factors is explained by the predictors.

## 4. Discussion

The current study aimed to examine the predictive relationships between character strength factors and *phronesis* motivation with regard to positive academic outcomes in a sample of Spanish undergraduates. The results showed that both dimensions of character—behavioural and motivational—were related to specific academic factors. The behavioural dimension of character, expressed through the character-strength factors, showed a positive association with academic engagement and performance. The motivational dimension of character, operationalized with the *phronesis* motivation factor, was positively related to academic engagement and negatively with career self-doubt. For the first time, the current study provides evidence in favour of the combined role of character-strength factors and *phronesis* motivation with regard to academic outcomes. Next, we offer a separate treatment for each of the specific goals of this research.

### 4.1. Characteristics of Participants Regarding Character and Academic Outcomes

The *first specific objective* of this study was to summarize the characteristics of participants regarding character and academic outcomes. Scalar scores of the included measures were mostly in line with previous research. These results give support to *Hypothesis 1* of the present study. With regard to character, participants tended to describe themselves as possessing more caring strengths (e.g., gratitude, love, kindness) than inquisitiveness or self-control strengths. This is in line with previous research on character strengths in Spanish university students [82]. Concerning the *phronesis* components, the majority of participants placed themselves between the middle and the high portion of their respective scales. This tendency was observed in past studies using the same emphatic concern and contingencies of self-worth measures [83,84]. The only difference with past studies was related to the moral identity scale. Teenagers in previous research have tended to place themselves in the mid-section of this scale [66], while current participants exhibited scores pointing mostly to the top part of the scale. This difference is thought to be related to the developmental stage of the current sample of emerging adult students when compared to the early and middle adolescents in that past study.

Participants in this research showed academic outcomes’ scores in line with previous research. Regarding academic engagement, participants showed higher scores on the dedication dimension of engagement in comparison with the vigour dimension. Similar results were reported in a previous study using the same scale [85]. In the case of career self-doubt, the current sample presented mid-to-low scalar scores. This finding is in line with previous research on vocational identity processes [28].

### 4.2. Character Strength Factors as Predictors of Academic Outcomes

The *second specific objective* in this study was to examine the predictive relationships between character-strength factors and the academic outcomes of engagement, career self-doubt and performance. We found that the three character-strength factors could be adequately modelled as predictors of academic outcomes. These findings give support to *Hypothesis 2* in the current study. The caring factor showed significant relationships with the vigour dimension of engagement, on the one hand, and with the dedication dimension of engagement and career self-doubt on the other. The positive association between the strengths that comprise caring and academic engagement was reported in previous research [25]. Notwithstanding, we propose separate interpretations for the two dimensions of engagement addressed in this study (vigour and dedication). Regarding vigour, we interpret its association with caring as stemming in part from the conceptual similarity between these two constructs. Vigour and caring included items that assessed positive emotionality: some referred to attending classes (vigour), and others to acknowledging things received in life (gratitude) or the consequences of being kind to others (kindness). A similar interpretation has been suggested for past research showing strong correlations between extraversion and subjective well-being [14] (p. 77).

In the case of dedication, and also for career self-doubt, we turn to insights from vocational psychology to interpret the present results. The items of the dedication subscale mainly refer to being enthusiastic about the chosen career. We find this dimension to have great connection to the vocational attitude of career commitment [86]. Thus, we interpret the correlation between caring and both dedication and career self-doubt as indicative of the beneficial role of cultivating positive relationships in career development. Past research has shown the positive contribution of attachment—a construct strongly connected to the character strength of love—[87,88] and kindness [89] to career commitment. Attachment has been also negatively correlated to career self-doubt or decision-making difficulties in previous research [34].

The other two character-strength factors of inquisitiveness and self-control were also associated with academic outcomes. Inquisitiveness showed itself to be related to academic performance. This result is in line with past research using character strengths individually [90] or compound strength factors [50]. Current findings and past studies converge in proposing intellectual strengths as good predictors of academic achievement when all the strengths are considered. In the case of the self-control factor, it showed a small correlation with the dedication dimension of engagement. The positive association between self-control strengths such as self-regulation and academic engagement has been reported in previous research [85]. Other constructs, conceptually close to self-control, have also shown correlations with academic engagement. This is the case of grit and conscientiousness [91], coping strategies [92,93] and resilience [92]. In accordance with the present results, it is possible to argue that caring and self-control are strengths more related to the emotional aspects of academic success (e.g., engagement), while inquisitiveness appeared to be associated with the adaptative behaviours (e.g., performance) expressing academic achievement.

### 4.3. The Role of Phronesis Motivation

The *third specific objective* of this research was to examine the changes produced in the predictive model when *phronesis* motivation was included. Using this construct as an additional predictor, we observed an important change in the pattern of predictive correlations. *Phronesis* motivation substituted caring in predicting both the dedication dimension of engagement and career self-doubt. With regard to the rest of the model, caring, inquisitiveness and self-control continued to be predictors of the vigour dimension of engagement, performance and dedication, respectively. These results partially support *Hypothesis 3. Phronesis* motivation was a significant predictor of two of the three academic outcomes in this study. Since this is the first study examining the role of *phronesis* motivation in academic outcomes, there is no previous empirical research for comparison. However, we interpret our findings in the light of the latest literature proposed for the *phronesis* construct [68]. According to such literature, *phronesis* should correlate with subjective measures of well-being. This is indirectly supported by the current findings, since we found *phronesis* motivation related to well-being related outcomes in the academic context. Regarding the specific academic outcomes predicted by *phronesis*, they were career-related factors. This seems compatible with the idea of *phronesis* as a meta-virtue that helps individuals to make decisions according to a blueprint of the good life [60]. In that blueprint, career aspirations are believed to be present, as our results suggest.

The inclusion of *phronesis* motivation produced an increase in the amount of explained variance of the academic outcomes’ factors. This finding gives support to *Hypothesis 4* in the current study. Regarding its interpretation, this result is thought to be a consequence of including both behavioural and motivational aspects of character as predictors of academic outcomes. An interesting aspect of this combined model is that the increment in explained variance was observed when *phronesis* replaced caring in predicting the career-related factors. With regard to this point, we postulate the following reading. We start by observing that caring and *phronesis* motivation showed a strong correlation. This suggests a conceptual similitude between the two constructs. However, when explained variances were examined, *phronesis* motivation predicted more variance than the caring factor. On the one hand, we interpret this finding as indicating that some aspects of career engagement require the cultivation of caring virtues. On the other hand, we argue that career engagement is not only dependent on caring behaviours, but also, and more strongly, on the internal moral (and at the same time pro-social) motivation of the agent. In other words, although cultivating positive relationships can certainly contribute to career-related positive emotions, the internal moral motivational aspects of character seem to be the most relevant predictors.

### 4.4. Limitations

There are some limitations in this research that have to be mentioned. First, the small sample size and the incidental non-probabilistic sampling design put limits on the generalizability of the findings. As a consequence, the patterns of associations described in this study are thought to characterize young university students from a particular cultural and socioeconomic background. In addition, the sample composition (more women than men) does not permit us to investigate gender-specific differences in the contribution of character to academic outcomes. Past studies have described gender differences in moral development (i.e., women showing higher levels of moral identity and moral reasoning in comparison to men [94,95,96]). However, it was beyond the scope of the present study to examine how such differences can modify the contribution of character to academic outcomes. Future studies with more diverse sample characteristics are necessary to replicate or contradict the tendencies we observed in the current research. Also, these studies could help in the exploration of the impact of gender differences in the contribution of character to academic outcomes.

A second limitation of the current research is related to its cross-sectional design. Based on our empirical data, it is not possible to infer the causal direction of the reported associations. Our models presumed that character traits and moral motivation were the influencing factors. However, it is plausible that the direction of the causal association can be modelled in a contradictory way: academic motivation and achievement as the cause of displaying high character traits. In fact, one of the current perspectives on the empirical study of character seems to follow this second explanatory direction [97]. More research is needed, using longitudinal and experimental designs, to help answer the question of the causal direction.

Regarding its level of analysis, a third limitation of the present study is that only individual variables were considered and not environmental ones. Contextual factors, such as classroom atmosphere, teaching style or faculty organization, were not taken into account. Previous research has suggested that contextual factors, such as self-regulatory teaching, can interact with individual personality traits in the prediction of engagement and burnout [98]. Future research is needed to examine the influence of such aspects, in particular, research into the factors that promote the practice of virtues as suggested in the latest studies on the measurement of virtues and character [61]. Lastly, a fourth limitation is that social desirability biases were not fully controlled. Participants were asked to complete anonymous surveys. This is thought to have reduced the tendency to overemphasize socially desirable traits in survey responses. Nevertheless, other mechanisms such as including a social desirability scale together with the measurement instruments were not applied in this research. Past studies have suggested that social desirability is not problematical in virtue-state reports [4] (p. 11). However, controlling for social desirability has been recommended to adequately separate character effects from the influence of such a bias factor in predicting outcomes of interest [4].

## 5. Conclusions

This research has examined the role of two dimensions of character (i.e., behavioural and motivational) in predicting specific academic outcomes of university students. The principal finding was that both dimensions of character showed associations with the different academic outcomes included in this study. For the first time, the current research has provided support for the contribution of the motivational aspects of character in the academic context of undergraduates. These results invite us to reflect on the importance of a holistic conception of character and virtue. In particular, they revealed that moral motivational aspects of character—the desire of being a moral person and empathic-concern emotions—appear to be connected to career-related engagement and the absence of career indecision. This key contribution is in line with the latest research bridging social science with moral philosophy in the domain of professional development [99,100,101]. Our findings corroborate the importance of moral motivation for academic and career success. This is a call to take into account not only behavioural but also moral motivational dimensions of character in the education of young adults and their preparation to become professionals. By preparing for and exercising their professions with a social responsibility sense, undergraduates and working professionals can contribute to the flourishing of themselves and society.

## Figures and Tables

**Table 1:** Bivariate correlations and descriptive statistics of character strengths, *phronesis* motivation and academic outcomes.

	1	2	3	4	5	6	7	8	9	10	11	12	13	14	15	M	SD	S	K
**Character Strengths**																			
1. Gratitude																4.24	0.60	−0.87	0.63
2. Love	0.60															4.00	0.85	−0.88	0.37
3. Kindness	0.53	0.51														4.42	0.55	−0.94	0.69
4. Perspective	0.24	0.20	0.19													3.85	0.75	−0.18	−0.60
5. Creativity	0.23	0.22	0.22	0.45												3.66	0.68	−0.20	−0.17
6. Love of learning	0.00	−0.01	−0.06	0.34	0.26											2.91	0.96	0.20	−0.65
7. Perseverance	0.18	0.20	0.21	0.24	0.25	0.09										3.65	0.80	−0.37	−0.05
8. Prudence	0.29	0.26	0.25	0.25	−0.00	0.07	0.35									3.85	0.73	−0.40	0.09
9. Self-regulation	0.07	0.08	0.21	0.18	0.12	0.18	0.35	0.29								3.16	0.80	−0.15	−0.17
***Phronesis* Motivation**																			
10. IRI Empathy	0.43	0.41	0.48	0.16	0.02	0.09	0.17	0.32	−0.01							4.04	0.63	−0.52	0.31
11. MSR Measure	0.42	0.28	0.38	0.14	−0.04	−0.05	0.08	0.13	−0.04	0.41						21.78	3.82	−0.34	−0.59
12. CSW Virtue	0.24	0.15	0.28	0.10	0.01	0.05	−0.00	0.07	−0.10	0.38	0.32					5.47	0.97	−0.40	−0.45
**Academic Outcomes**																			
13. Vigour	0.28	0.24	0.24	0.23	0.23	0.19	0.18	0.15	0.14	0.20	0.18	0.29				3.45	1.22	−0.29	−0.24
14. Dedication	0.24	0.15	0.25	0.18	0.06	0.14	0.23	0.23	0.07	0.29	0.22	0.29	0.50			4.87	1.01	−10.36	20.92
15. Career Self-Doubt	−0.17	−0.18	−0.15	−0.12	0.10	−0.13	−0.03	−0.07	−0.12	−0.29	−0.19	−0.20	−0.30	−0.50		2.63	0.76	0.20	−0.31
16. Performance	0.11	0.01	−0.05	0.07	0.07	0.32	0.15	0.10	0.08	0.07	0.07	0.10	0.30	0.22	−0.11	4.73	1.03	0.42	0.41

Bivariate correlations greater than ∣0.15∣ were significant at *p <* 0.05, and those greater than ∣0.20∣ were significant at *p <* 0.01; IRI = Interpersonal Reactivity Index; MSR = Moral Self-Relevance; CSW = Contingencies of Self-Worth; S = Skewness; K = Kurtosis.

**Table 2:** Goodness of Fit of SEM models. Character Strength factors (CS) and *Phronesis* Motivation (PM) predicting Academic Outcomes (AO).

Model	Description	*χ* ^2^	*df*	CFI	TLI	RMSEA	SRMR
M1	3 CS factors predicting 4 AO factors (all regression paths)	220.20	169	0.95	0.94	0.04	0.06
M2	3 CS factors predicting 4 AO factors (5 regression paths)	225.22	176	0.95	0.94	0.04	0.06
M3	3 CS and 1 PM factors predicting 4 AO factors (all regression paths)	307.88	225	0.93	0.91	0.05	0.06
M4	3 CS and 1 PM factors predicting 4 AO factors (5 regression paths)	315.80	236	0.93	0.92	0.04	0.06

The AO factors included Academic Engagement (Vigour and Dedication), Career Self-Doubt, and Performance.

**Table 3:** SEM regression paths of Character Strength factors and *Phronesis* Motivation predicting Academic Outcomes.

	Character Strength Factors Model (M2)							Character Strength Factors + Phronesis Model (M4)						
					95% CI							95% CI		
Outcomes	Predictors	Est.	SE	Std. Est.	L	U	R^2^	Predictors	Est.	SE	Std Est.	L	U	R^2^
Eng. Vigour	Caring	1.07	0.23	0.40 **	0.24	0.57	0.16	Caring	1.16	0.23	0.43 **	0.28	0.58	0.19
Eng. Dedication	Caring	0.54	0.24	0.23 *	0.03	0.43	0.14	Phronesis	0.79	0.26	0.35 **	0.17	0.53	0.21
	Self-control	0.43	0.26	0.20	−0.04	0.44		Self-control	0.44	0.23	0.21 *	0.00	0.42	
Career Self-Doubt	Caring	−0.46	0.17	−0.26 **	−0.43	−0.09	0.07	Phronesis	−0.72	0.19	−0.42 **	−0.59	−0.26	0.18
Performance	Inquisitiveness	0.44	0.14	0.26 **	0.11	0.41	0.07	Inquisitiveness	0.45	0.15	0.27 **	0.12	0.41	0.07

Notes: Eng. = Engagement; ** *p* < 0.01; * *p* < 0.05.

## Data Availability

The data is not public for data protection reasons. They can be obtained by contacting the first author of the article.
